# Supplementary material for: School bullying and its association with psychological wellbeing: Findings of the Fujian Adolescent Mental Wellness Study (FAMWeS)
Source: PLoS One. 2026 Apr 16;21(4):e0315993. doi: 10.1371/journal.pone.0315993 (PMC13086429; doi:10.1371/journal.pone.0315993)
Supplement: Attachment — Submitted filename: Response to Reviewers.doc [file pone.0315993.s005.doc]

**Response to Reviewers**

**Required Revisions (Minor)**

**Requirements 1:**

Clarify analytic approach regarding cluster sampling
Although the sampling strategy employed a multistage cluster design, the regression analyses do not appear to adjust for clustering at the school/class level.
Please explicitly acknowledge this in the Limitations section, noting the potential for underestimated standard errors.

Response:

We thank the reviewer for highlighting this important methodological concern. We agree that failing to adjust for clustering effects in a multistage cluster sampling design may lead to underestimated standard errors and affect the precision of statistical inferences. In response, we have added the following statement to the **Limitation section (Section 6)**:

“It should also be noted that we did not adjust for clustering effects at the school/class level in our regression analysis, which may have led to an underestimation of the standard error.”

**Requirements 2:**
Tidy remaining editorial elements
A few minor textual issues remain (e.g., repeated lines in the Data Availability statement and minor typographical inconsistencies).
Please revise for clarity and consistency.
Response:
We thank the reviewer for their careful reading. We have thoroughly reviewed the manuscript and made the following corrections:

**Data Availability Statement**: Duplicate lines have been removed to ensure clarity and conciseness.
**Typographical inconsistencies**: We have corrected punctuation, spacing, capitalization, and other minor formatting issues throughout the text.
**Terminology consistency**: We have ensured that the terms “bullying victimization” and “bullying behavior” are used consistently across the manuscript (as previously suggested by reviewers).

**Requirements 3:**
Optional but recommended:
Briefly reinforce in the Limitations that teacher-administered questionnaires may increase social desirability bias in sensitive topics (bullying, mental health).

Response:
We thank the reviewer for this valuable suggestion. We have added the following statement to the **Limitations section (Section 6)：**

“Additionally, the administration of questionnaires by classroom teachers may have influenced the honesty of students’ responses, particularly on sensitive topics such as bullying.”
