# Supplementary material for: School bullying and its association with psychological wellbeing: Findings of the Fujian Adolescent Mental Wellness Study (FAMWeS)
Source: PLoS One. 2026 Apr 16;21(4):e0315993. doi: 10.1371/journal.pone.0315993 (PMC13086429; doi:10.1371/journal.pone.0315993)
Supplement: Attachment — Submitted filename: Response_to_Reviewers_auresp_3.doc [file pone.0315993.s006.doc]

**Response to Reviewers**

**Required Revisions (Minor)**

**Requirements 1:**

Sample size consistency
Please reconcile the discrepancy in the reported sample size between the Abstract and the Results/Tables. If exclusions were applied after initial screening, clearly state this and ensure the same final N is used consistently throughout the manuscript.

Response:

A total of 54,368 questionnaires were initially collected. During the data cleaning stage, we applied predefined quality control criteria (including trap question screening and removal of straight-lining responses) to exclude invalid questionnaires. Consequently, the final analytic sample comprised 53,157 valid responses, which is the sample size used in all tables (Table 1 and Table 2).

To ensure consistency, we have made the following revisions:

**1. Abstract**: Changed “Analysis of 54,368 responses” to “Analysis of 53,157 valid responses” to reflect the final analytic sample.

**2. Results, first paragraph**: Changed “A total of 54,368 complete responses were included” to “A total of 54,368 questionnaires were collected. After data cleaning, 53,157 valid responses were included in the final analysis.”

**3. Table titles**: Table 1 and Table 2 now clearly indicate N=53,157, consistent with the text.

**4. Other descriptions**: All mentions of sample size throughout the manuscript have been reviewed and revised to consistently use the final analytic sample of **53,157**.

**Requirements 2:**
Consistency between text and tables
Carefully review the Results section to ensure that all odds ratios, confidence intervals, and p-values reported in the text exactly match those presented in the corresponding tables. Any discrepancies should be corrected.
Response:
Thank you for your careful review regarding the consistency between the text and the tables. We acknowledge that there were discrepancies in the reporting of some data when citing from the tables into the text, which occurred due to clerical errors. We have now thoroughly verified all data points and made the necessary revisions to ensure that all odds ratios, confidence intervals, and p‑values reported in the text are fully consistent with those presented in the corresponding tables. All modifications have been marked using track changes in the revised manuscript for your reference. We appreciate your attention to detail and apologize for any confusion caused.

**Requirements 3:**
Data Availability statement
Please revise the Data Availability statement to align fully with journal policy by specifying a clear institutional access pathway for data requests. Avoid phrasing such as “available from the authors upon request” and instead indicate the responsible institutional body and conditions for access.

Response:
Thank you for the guidance on revising the Data Availability statement to align with PLOS ONE policy. We have modified the statement as follows to ensure it specifies a clear institutional access pathway:

“Due to ethical and confidentiality concerns regarding adolescent participants, the research data are not publicly available. Data requests may be submitted to the Ethics Committee of Fuzhou University Affiliated Provincial Hospital (Email: fjslkyk@163.com), which will review requests in accordance with institutional ethical and privacy policies. Access will be granted to qualified researchers for purposes aligned with the study’s ethical approval.”

**Requirements 4:**

Minor editorial and formatting issues
Perform a final careful language and formatting check to correct minor typographical errors, spacing inconsistencies, and ensure uniform presentation of abbreviations, percentages, and decimal places across the manuscript and tables.

Response:

Thank you for your careful review of the editorial and formatting details. We have conducted a thorough language and formatting check throughout the manuscript. All revisions have been marked using track changes in the revised manuscript for your reference. We believe that the language and formatting of the manuscript now fully meet the journal’s requirements. Thank you again for your meticulous review.
