# Supplementary material for: School bullying and its association with psychological wellbeing: Findings of the Fujian Adolescent Mental Wellness Study (FAMWeS)
Source: PLoS One. 2026 Apr 16;21(4):e0315993. doi: 10.1371/journal.pone.0315993 (PMC13086429; doi:10.1371/journal.pone.0315993)
Supplement: Attachment — Submitted filename: Reviewer comment PONE-D-24-55844.docx [file pone.0315993.s002.docx]

Overall

The article is well-written. However there are some minor wording mistakes in the results section.

Abstract:

Objective, methods and results were clearly written in the abstract.

Introduction:

Well written.

Methods:

Methods were described in detail.

Results:

Results were reported in detail. However there are some wording mistakes:

1. Under 3.2 Bullying Behavior, last line in paragraph 2:

“Based on academic achievement, the odds of **experiencing bullying** were 1.22 times higher for participants with D-level performance (OR 1.22, 95% CI 1.10 1.34) and 1.40 times higher for those with E-level performance (OR 1.40, 95% CI 1.25-1.57) compared to those in the top 15% (A level).”

2. Under 3.2 Bullying Behavior, last line in paragraph 4:

“Individuals with a GHQ score of 3-12 had 1.71 times higher odds of **bullying victimization** compared to those with a score of 0-2 (OR 1.71, 95% CI 1.60-1.83).”

Comment: Author used “experiencing bullying” and “bullying victimization” as the variable but the result is about bullying behavior not bullying victimization.

3. Added suggestion: In 3.3 Feeling Safe in School, author describes table 2 showing the association between bullying victim/behavior and feeling safe. However, the table also showed many other important factors. Suggest changing the sentence to: “The associated factors with feeling safe in school were shown in Table 2”

Discussion:

Well written.
